# Opportunities and Barriers to Innovation in Acute Care Pediatrics

**DOI:** 10.3389/fped.2020.00043

**Published:** 2020-02-20

**Authors:** Lakhmir S. Chawla

**Affiliations:** Department of Medicine, Veterans Affairs Medical Center, San Diego, CA, United States

**Keywords:** acute care pediatrics, investment, academic incentive, ROI, clinical trials network

## Introduction

Drug development is largely an exercise in failure punctuated with infrequent, and rarely, dramatic successes. In general, only 1 in 10,000 compounds assessed for medical use go on to become Food and Drug Administration (FDA) approved drugs (1). This attrition rate is exacerbated by the fact that drug development is usually a long-term project. In general, the time from initial compound development to FDA approval requires 10–15 years. Added to the poor success rate and long duration, drug development requires compounds that are produced under good manufacturing practice (GMP), requires animal and toxicology studies, that must conform to good laboratory practice (GLP), and require successful clinical studies in patients that conform to good clinical practice (GCP). All of these provisions add cost and time to the process. On average, the development cost for a drug which eventually gains FDA approval is $30–150 million (2). Thus, it is clear that drug development is capital intensive and requires both patience and perseverance to achieve success.

With this background in mind, recognize that the capital-intensive portion of this equation sets the stage for which therapeutic areas are pursued and which are ignored. Investors do not mind long-term investments so long as they achieve a good return on the investment (ROI). Why does an investor choose drug development as an investment over something more banal such as purchasing the stock of a large stable company (i.e., a large utility company)? The answer lies in the ROI—better ROI justifies taking more risk. The companies that decide on which therapeutic areas to pursue are cognizant of these factors and tend to pursue drugs that will generate a good ROI; thus, cultivating the requisite investment.

These factors conspire to make pediatric acute care medicine less attractive than other therapeutic indications. For purposes of this exercise, we will use a 20-year timeline which assumes 10 years to drug approval (a rapid time frame to get a drug approved) and 10 years to make profits and recoup the investment. In order to illustrate this point, let's look at a conservative investment of 100 million dollars in a safe high-yielding utility stock that generates a reliable 4% annual dividend (assume annual compounding of the interest) and assume that the stock price does not change for 20 years. At the end of 20 years, an investor who invested in this utility company would have 219 million dollars. Therefore, in order for an investor to decide to place their investment in drug development instead, the investor expects a better ROI. In terms of investment, any factor which increases the time of drug development, increases the risk of failure, or decreases the ability to recoup profits will make an investment less attractive. The question at hand is simply this: does acute care pediatrics increase the time of development, have a higher risk of failure, and does it decrease profit potential? The answer is yes on all counts.

## Pediatric Research and Impact on the Finances of Development

**Figure 1:**
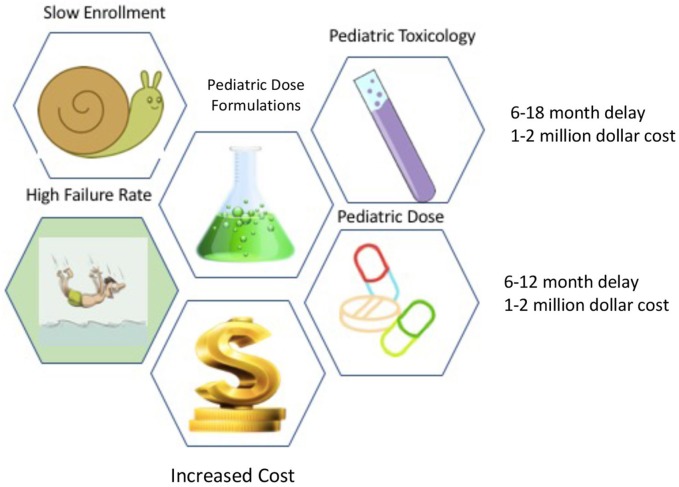
Children versus Adult Development Hurdles.

How does pediatric acute care decrease the *expected* ROI. First, in order to bring a drug into Phase 1 studies (humans), the drug must undergo comprehensive toxicology studies in two species of animals (there can be exceptions, but this the standard guidance). Any drug destined for investigation in children requires juvenile toxicology studies which add both time and money to the equation. In addition, pediatric trials have a higher failure rate than adult studies and they take longer to enroll. Moreover, since children are generally in good health, there are fewer sick patients available to study. Studies in in children typically require different dose formulations, and drug production work of these smaller formulations need to be done before pivotal trials can be done. If these hurdles were not enough, drug companies make more money on outpatient drugs than acute care drugs. One reason for this is that acute care is shorter in duration, whereas in chronic diseases the drugs are taken for a longer duration of time (i.v. antibiotics vs. statins) (Figure 1).

For purposes of investment decision making, let's assume the average ROI in a portfolio of drugs is 8% (double the safe utility company) and assume the stock price does not change and drug is guaranteed to succeed. Now if we take the same 100-million-dollar investment and change the ROI from 20 years and add a 5-year delay for acute care pediatrics the value proposition is quite different. In the first case of a 20-year timeline, 100 million dollars invested at 8% pays off 466 million. If that same 466 million was earned over 25 years instead of 20, the interest rate return is 6.35% instead of 8%—a difference of 1.65% or 165 basis points. This may not seem like a big difference, but if it was your own mortgage payment, that difference would be meaningful. For an investor with 100 million dollars, the difference is much larger. Assessed another way, 100 million dollars invested over 20 years at 6.35% yields 342 million whereas 8% yields 466 million for a difference of 124 million dollars. If it was your money, would you invest in the acute care pediatric drug, or the drug for adult type II diabetics? Since most investors are capitalists, they tend to invest in chronic prevalent adult disease and avoid acute diseases.

The FDA is aware of these issues and has attempted to incentivize pediatric development. If a drug gains a pediatric indication after the initial adult indication, the exclusivity of the drug is increased by 6 months. The FDA has also placed incentives such as priority review vouchers to incentivize pediatric research on drug development on rare diseases. Nonetheless, the brutal truth is this—acute care pediatrics is a higher risk investment with worse ROI compared to adults with a chronic disease. In my view, these are key factors that drive the general neglect felt by most acute care physicians particularly pediatricians.

## Discussion

What can be done to mitigate these factors? Simply put, the acute care pediatric community can only directly control one of these many factors. The ability to recoup profits from the chronic outpatient vs. the acute inpatient is a structural issue and will require policy initiatives. However, the probability and time to success can be improved. In order to improve the time to completion of acute care pediatric studies, I would offer four suggestions (Table 1). One, clinical trial networks involving many pediatric-centered hospitals must be organized and maintained so that when a drug is ready to be tested, the infra-structure is already in place. Two, screening tools that leverage electronic medical records in order to facilitate timely and efficient enrollment of patients should be put into place as a part of routine practice. Third, the acute care pediatric community must embrace precision diagnostics into their regular clinical practice; thus enabling identification of patients that may benefit from an investigational drug. All too often, the nihilistic view is that since there is no drug for this disease, I don't need to diagnose it in a precise or timely fashion and these diagnostics are not put into routine practice. Fourth, academic centers must create financial and promotion incentives to support clinical investigators so that the talent is brought to the bedside and not pushed into the lab. Incentives matter, and currently the incentives at academic centers is to get government and non-profit research to increase the coveted indirect funding dollars and avoid industry trials. Indirect funding is good for an academic center's bottom-line, but in order to gain FDA approval, the sponsor must be a company that can make the drug to the FDA standard. The National Institutes of Health, Gates Foundation, and other luminary institutions do not manufacture drugs even though they may fund billions in research dollars. Plainly stated, if the acute care pediatric care community wants to alter the financial equation and shepherd investment into this therapeutic area, they must enroll their trials faster and cheaper while maintaining high quality and safety.

**Table 1:** Strategies to improve enrollment time and efficiency.

**Strategic Investment**	**Reasons**
Standing acute care pediatric trial networks	Avoids the feast/famine of investigator and coordinator personnel and budgets
Enrollment Screening Tools that are linked to the EMR	Improve efficiency of planning trials and enrollment
Use of precision diagnostics	The routine availability of these diagnostics makes them actionable for study enrollment. The use of these diagnostics for research purposes only increases cost and time
Alignment of incentives	Promotion and academic advancement must align with the needs of improvements in clinical trial enrollment and efficiency. The failure to align these incentives precludes the ability to retain talent.

In conclusion, the nature of pediatric acute care drug development creates a tendency for under-investment. Some of the factors that contribute to this are structural and hard to change. However, initiatives that foster collaboration, academic promotion incentives for investing in clinical trials personal/infra-structure, and improved trial enrollment may help offset these hurdles.

## Author Contributions

The author confirms being the sole contributor of this work and has approved it for publication.

### Conflict of Interest

LC is the Chief Medical Officer of La Jolla Pharmaceutical Company. The opinion offered is LC's personal opinion and does not represent the opinion of La Jolla Pharmaceutical Company, its employees, or board members.
